# Anti-SSA/Ro60kd Antibodies Are Associated With Positive Salivary Gland Biopsy in Sjögren’s Syndrome: A Retrospective Exploratory Study

**DOI:** 10.7759/cureus.107696

**Published:** 2026-04-25

**Authors:** Rita Matos Sousa, Marta Duarte, Ana Filipa Martins, Carlos Capela

**Affiliations:** 1 Clinical Sciences, Escola de Medicina, Universidade do Minho, Braga, PRT; 2 Internal Medicine, Unidade Local de Saúde de Braga, Braga, PRT; 3 Clinical Research, Centro Clínico Académico - Braga, Associação (2CA-Braga), Braga, PRT; 4 Clinical Sciences, Life and Health Sciences Research Institute (ICVS), School of Medicine, University of Minho, Braga, PRT; 5 Clinical Sciences, Life and Health Sciences Research Institute (ICVS)/3B's Portuguese Government Associate Laboratory, Braga, PRT; 6 Internal Medicine, Hospital de Braga, Braga, PRT

**Keywords:** anti-ssa ro60kd, autoimmune disease, histopathology, minor salivary gland biopsy, sjögren’s syndrome

## Abstract

Introduction/objectives: Sjögren’s syndrome (SS) is a systemic autoimmune disease with heterogeneous clinical, laboratory, and histopathological manifestations, which complicate timely diagnosis. Minor salivary gland biopsy (MSGB) is a key component of SS classification, although its relationship with clinical and serological features remains incompletely characterized. This study aimed to evaluate the association between MSGB findings and clinical-laboratory variables in patients with SS and to identify variables associated with a positive biopsy.

Methods: We conducted a retrospective observational study at Braga Local Health Unit, including 37 patients diagnosed with SS between 1995 and 2024 who underwent MSGB. Patients were categorized into group A (positive biopsy, n = 17) or group B (negative biopsy, n = 20). Clinical, serological, and histopathological variables were analyzed using Fisher’s exact test and multivariate logistic regression.

Results: Thirty-seven patients were included (17 biopsy-positive, 20 biopsy-negative). The most frequent manifestations were ocular dryness (91.9%), oral dryness (78.4%), anti-SSA antibodies (Ro52: 51.4%; Ro60 kDa: 54.1%), antinuclear antibodies (ANA) titers >1:160 (64.9%), rheumatoid factor (RF) positivity (40.5%), elevated erythrocyte sedimentation rate (ESR) (67.6%), and arthralgia (56.8%). Fisher’s exact test identified several statistically significant associations between clinical and serological variables. In multivariate logistic regression, anti-SSA Ro60 kDa positivity was independently associated with a positive biopsy result (OR: 12.94; 95% CI: 1.39-120.13; p = 0.024), while myalgias were associated with a lower likelihood of biopsy positivity.

Conclusion: This exploratory study identified associations between serological markers and salivary gland biopsy findings in SS. Anti-SSA Ro60 kDa positivity may be associated with histopathological involvement; however, given the small sample size and wide confidence intervals, these findings should be interpreted cautiously and require validation in larger prospective studies.

## Introduction

Sjögren’s syndrome (SS) is a systemic autoimmune disease characterized by lymphocytic infiltration of exocrine glands, primarily salivary and lacrimal glands, leading to glandular dysfunction and sicca symptoms [1-3]. It mainly affects females (female-to-male ratio ranging from 9:1 to 14:1), typically diagnosed between the fourth and sixth decades of life, with a prevalence estimated to be around 0.1-4.8% [1,4,5]. While SS may present as a primary disease or secondary to other autoimmune conditions, such as rheumatoid arthritis (RA) or systemic lupus erythematosus (SLE), both forms share a broad spectrum of systemic manifestations, including musculoskeletal, pulmonary, neurological, and hematological involvement, amongst others [2,6-13].

The pathogenesis of SS involves genetic predisposition (notably HLA-DR3 and DR4), environmental triggers, and chronic immune activation [5,14,15]. Histopathologically, SS is characterized by periductal lymphocytic infiltrates (predominantly CD4+ T cells), acinar atrophy, and ductal hyperplasia, leading to subsequent glandular dysfunction [16-18]. These infiltrates are often accompanied by the production of autoantibodies, particularly anti-SSA/Ro and anti-SSB/La, which have been associated with earlier disease onset, parotid enlargement, and more severe extraglandular manifestations [6,18]. Interestingly, anti-SSA/Ro antibodies can also appear years before clinical symptoms [19,20].

Despite advances in understanding SS pathogenesis, diagnosis remains difficult because of the variety of clinical symptoms and the absence of a single definitive test [21]. The current American College of Rheumatology (ACR)/European Alliance of Associations for Rheumatology (EULAR) classification criteria depend on a combination of clinical, serological, and histopathological findings, with minor salivary gland biopsy (MSGB) serving as a key component [22]. However, a significant number of patients do not meet formal criteria despite having evident clinical disease, and MSGB interpretation can be hindered by variability in histological reporting and sampling [23,24].

Prior studies have suggested that specific serological markers, such as anti-SSA/Ro antibodies, correlate with disease severity and MSGB findings; however, the strength of these associations remains uncertain, particularly regarding the predictive value of specific autoantibody subtypes (e.g., anti-SSA Ro52 vs. Ro60kd) [18,24]. Furthermore, the relationship between biopsy results and systemic manifestations remains unclear [24].

Given the complexity of SS diagnosis and the heterogeneity of clinical, laboratory, and histopathological findings, further understanding of the relationships between these domains is warranted. Therefore, this study aimed to explore the association between MSGB findings and clinical and serological variables in patients with SS, with the objective of generating hypotheses regarding potential predictors of biopsy positivity.

## Materials and methods

Study design and participants

We conducted a retrospective, observational study at Braga Local Health Unit (ULS Braga). Patients were diagnosed with primary or secondary SS by experienced physicians at the Autoimmune Diseases Outpatient Clinic of the Internal Medicine Service from January 1995 to December 2024 based on accepted classification criteria in use at the time of diagnosis. Primary and secondary forms were distinguished according to the presence of concomitant autoimmune diseases. Given the long inclusion period (1995-2024), diagnostic criteria evolved over time; however, all cases were reviewed to confirm consistency with contemporary clinical standards, thereby minimizing diagnostic heterogeneity.

Group classification

Patients were divided into two groups based on MSGB results: group A - positive biopsy (histological findings consistent with SS); group B - negative biopsy (findings not consistent with SS).

Data collection and variables

Clinical, laboratory, and histopathological data were gathered from electronic medical records (Glintt® platform). Variables included age, sex, presence of autoimmune diseases, sicca symptoms (oral and ocular dryness), systemic manifestations (e.g., arthralgia, myalgia, and Raynaud’s phenomenon), serological markers (anti-SSA/Ro52, anti-SSA/Ro60kd, anti-La/SSB antibody titers, anti-nuclear antibodies (ANA) titers, rheumatoid factor (RF), complement levels, erythrocyte sedimentation rate (ESR), β2-microglobulin), and MSGB findings. All variables were analyzed as categorical and coded in a binary manner (present/absent). Continuous laboratory variables were categorized according to standard clinical thresholds.

Given the retrospective nature of the study, the availability of an MSGB report was used as the primary inclusion criterion to ensure diagnostic consistency. Clinical and serological variables were extracted when available. Missing data were handled using complete-case analysis. The proportion of missing data varied across variables due to the retrospective design and was generally low for key variables included in the main analyses. No imputation was performed to avoid introducing additional assumptions. This approach may have reduced the effective sample size and introduced bias, which is acknowledged as a limitation.

Histopathological assessment

MSGB specimens were obtained as part of routine clinical care. A biopsy was considered positive when histopathological findings were reported as consistent with SS, typically corresponding to focal lymphocytic sialadenitis. When available, a focus score ≥1 focus per 4 mm² (defined as aggregates of ≥50 lymphocytes) was considered indicative of a positive result. Due to the retrospective design and long inclusion period (1995-2024), histopathological reports were not fully standardized. Adequacy criteria (e.g., number of glands sampled or total glandular surface area) and exact focus scores were not consistently available. Biopsy interpretation was performed by pathologists as part of routine clinical practice, and no centralized or blinded review was conducted.

Ethical considerations

The study was approved by the Ethics Committee for Health of ULS Braga (CEULSB 81_2025), the University of Minho Life and Health Sciences Ethics Committee (CEICVS 028-2025), and the Institutional Data Protection Officer (20250028_MedInterna170225). The study was conducted in accordance with the Declaration of Helsinki and the General Data Protection Regulation (GDPR, EU 2016/679).

Statistical analysis

Descriptive statistics were presented as absolute frequencies and percentages. Group comparisons were conducted using Fisher’s exact test and the chi-square test. Sensitivity and specificity values for clinical variables were estimated in an exploratory manner, with MSGB as the reference. Effect sizes were evaluated using the phi coefficient and Cramer’s V.

Continuous variables were dichotomized according to clinically established reference thresholds to ensure consistency in interpretation across the extended study period. We acknowledge that this approach may reduce statistical power and obscure potential dose-response relationships.

Given the limited sample size, no stratified or sensitivity analyses were performed according to disease subtype (primary vs. secondary SS) or diagnostic era. These factors may contribute to heterogeneity and are acknowledged as limitations.

An initial multivariate logistic regression model, including clinically relevant variables, was performed. However, due to instability related to the small sample size and number of predictors, a reduced model was constructed. Variable selection in the final model was based on a combination of clinical relevance and statistical stability (e.g., exclusion of variables with extreme standard errors or implausible estimates).

Results were reported as odds ratios (OR) with 95% confidence intervals (CI). Statistical significance was set at p < 0.050. All analyses were performed using IBM SPSS version 29 (IBM Corp., Armonk, NY).

## Results

Population characteristics

A total of 37 patients with SS were included, of whom 33 (89.2%) were females. The most common age at diagnosis was 51-65 years (43.2%). Based on MSGB results, 17 patients (45.9%) were classified as group A (positive biopsy), and 20 (54.1%) as group B (negative biopsy) (Table 1).

**Table 1:** Demographic characteristics of the study population.

	Group A (n = 17)	Group B (n = 20)	Total (n = 37)
Gender			
Female	16/17 (94.1%)	17/20 (85.0%)	33/37 (89.2%)
Male	1/17 (5.9%)	3/20 (15.0%)	4/37 (10.8%)
Age at diagnosis			
0-20 years	0/17 (0%)	0/20 (0%)	0/37 (0%)
21-30 years	1/17 (5.9%)	1/20 (5.0%)	2/37 (5.4%)
31-40 years	3/17 (17.6%)	1/20 (5.0%)	4/37 (10.8%)
41-50 years	6/17 (35.3%)	7/20 (35.0%)	13/37 (35.1%)
51-65 years	5/17 (29.4%)	11/20 (55.0%)	16/37 (43.2%)
≥66 years	2/17 (11.8%)	0/20 (0%)	2/37 (5.4%)

In group A, the most common age range at diagnosis was 41-50 years (35.3%), while in group B, it was 51-65 years (55.0%). Overall, 43.2% (n = 16/37) of patients were diagnosed between the ages of 51 and 65 years, followed by the 41-50 years age group, which accounts for 35.1% (n = 13/37). The frequency of clinical and laboratory variables was assessed in both groups (Appendices).

When analyzing selected laboratory markers, anemia was observed in 24.3% of patients, and leukopenia in 29.7%. Complement C3 consumption was reported in 29.4% of group A and 21.6% of the total cohort, while C4 consumption was rare (2.7%, one patient from group B). Elevated RF was present in 58.8% of group A, representing 40.5% of all cases. Increased β₂-microglobulin and elevated IgG were observed in 23.5% of group A patients for both markers. ANA titers >1:160 were found in 82.3% of group A and 64.9% of the entire cohort.

Regarding autoantibodies, anti-La/SSB positivity was similar in both groups at 35.1% overall. Anti-SSA/Ro52 and anti-SSA/Ro60kd were found in 64.7% of group A patients, accounting for 51.4% and 54.1% of the total cohort, respectively. ESR was elevated in 67.6% of patients, with similar rates across both groups.

Regarding autoimmune comorbidities, 41.2% of patients in group A had at least one associated condition, compared to 25.0% in group B. In group A, the most frequent comorbidity was Hashimoto’s thyroiditis (23.5%), followed by psoriasis (5.9%) and sarcoidosis (5.9%). In group B, Hashimoto’s thyroiditis was also the most common (15.0%, 3/20), followed by systemic lupus erythematosus (5.0%, 1/20) and rheumatoid arthritis (5.0%, 1/20). No cases of lymphoma were identified from diagnosis through the time of data analysis.

Among clinical symptoms, the most common were ocular dryness (91.9%), oral dryness (78.4%), arthralgia (56.8%), parotid gland enlargement/parotitis (35.3% in group A only), paresthesias (29.7%), myalgias (27.0%), atrophic gastritis (18.9%), respiratory symptoms (cough and/or dyspnea, 18.9%), fatigue (16.2%), headaches (16.2%), and Raynaud’s phenomenon (13.5%). Oral dryness occurred in 82.4% of group A patients; ocular dryness in 94.1%. Paresthesias, headaches, myalgias, and arthralgia were seen in both groups. Parotid gland enlargement was only reported in group A.

An exploratory calculation of sensitivity and specificity was performed for individual clinical and laboratory variables, using MSGB results as the reference. These measures aimed to identify potential associations rather than to evaluate diagnostic test performance (Appendices).

Only one patient with a negative biopsy had a positive ocular staining test (≥5 or van Bijsterveld score ≥4 in at least one eye), and this patient also tested positive on the Schirmer’s test (≤5 mm/5 min in at least one eye). One other biopsy-negative patient underwent Schirmer’s testing with a negative result, while one biopsy-positive patient had a positive Schirmer’s test.

Correlation analysis between variables

Statistically significant and marginally significant associations observed in group A are summarized in Table 2. A marginally significant association was found between RF positivity and ANA >1:160 (p = 0.051), with a very strong magnitude of association (phi = 0.553, p = 0.023). A statistically significant and very strong association was observed between RF positivity and anti-SSA/Ro52 positivity (p = 0.035, phi = 0.663, p = 0.009). Similarly, anti-SSA/Ro52 positivity was strongly associated with ANA >1:160 (p = 0.029, phi = 0.627, p = 0.010), and with parotid gland enlargement (p = 0.043, phi = 0.545, p = 0.025).

**Table 2:** Significant correlations in group A (positive biopsy). ANA, antinuclear antibodies; RF, rheumatoid factor.

Variables	Test	p-value	Phi coefficient	p-value (phi)
RF positivity vs. ANA >1:160	Fisher’s exact test	0.051	0.553	0.023
RF positivity vs. anti-SSA Ro52 positivity		0.035	0.663	0.009
Anti-SSA Ro52 positivity vs. ANA >1:160		0.029	0.627	0.010
Anti-SSA Ro52 positivity vs. parotid gland enlargement		0.043	0.545	0.025

No statistically significant associations were found between the following variable pairs: RF positivity and arthralgia; anti-SSA/Ro52 positivity and anemia; anti-SSA/Ro60kd positivity and leukopenia; RF positivity and ANA >1:160; RF positivity and anti-SSA/Ro60kd positivity; and anti-SSA/Ro60kd positivity and ANA >1:160.

In group B, several associations were observed that were either statistically significant or marginally significant (Table 3). A marginally significant link was noted between ocular and oral dryness (p = 0.053), with a very strong strength of association (phi = 0.577, p = 0.010). A statistically significant and very strong association was found between anti-SSA/Ro60kd positivity and anemia (p = 0.050; phi = 0.504, p = 0.024). Additionally, RF positivity and anti-SSA/Ro52 positivity showed a statistically significant and very strong association (p = 0.040; phi = 0.707, p = 0.002).

**Table 3:** Significant correlations in group B (negative biopsy). RF, rheumatoid factor.

Variables	Test	p-value	Phi coefficient	p-value (phi)
Oral dryness vs. ocular dryness	Fisher’s exact test	0.053	0.577	0.010
Anti-SSA/Ro60kd positivity vs. anemia		0.050	0.504	0.024
RF positivity vs. anti-SSA/Ro52 positivity		0.004	0.707	0.002

No statistically significant associations were found between the following variable pairs: RF positivity and arthralgia; anti-SSA/Ro52 positivity and anemia; anti-SSA/Ro60kd positivity and leukopenia; RF positivity and ANA >1:160; RF positivity and anti-SSA/Ro60kd positivity; and anti-SSA/Ro60kd positivity and ANA >1:160.

Predictive analysis of salivary gland biopsy results

A multivariate logistic regression analysis was conducted using the most common independent variables observed in patients (RF positivity, ANA >1:160, anti-La/SSB positivity, elevated ESR, anti-SSA/Ro52 and Ro60kd positivity, oral and ocular dryness, paresthesias, headaches, parotid gland enlargement, myalgias, and arthralgia), with biopsy result as the dependent variable. In the initial regression, no statistically significant results were found, likely due to the large number of variables included.

To address this, a subset of variables was selected, including anti-SSA/Ro60kd positivity, anti-La/SSB positivity, oral dryness, headaches, and myalgias, while excluding variables with standard error >1000, extreme Exp(B) values, or excessively wide or infinite 95% confidence intervals. In the revised multivariate logistic regression model, 35.2% of the variability in biopsy results (Nagelkerke R² = 0.352) was explained by the included variables (p = 0.046).

Specifically, anti-SSA/Ro60kd positivity emerged as independently associated with positive biopsy (Wald = 5.07, OR: 12.94, p = 0.024). The presence of myalgias was linked to a lower likelihood of a positive biopsy (p = 0.020; B = -2.973), while the presence of headaches was of borderline statistical significance (p = 0.053) (Table 4). However, the wide confidence intervals observed indicate substantial uncertainty in the estimates, likely reflecting the limited sample size.

**Table 4:** Multivariate logistic regression analysis: predictors of positive salivary gland biopsy. S.E., standard error; CI, confidence interval.

Variable	B	S.E.	Wald	df	p-value	Exp(B)	95% CI for EXP (B)	
							Lower	Upper
Anti-La/SSB positivity	-1.422	1.028	1.915	1	0.166	0.241	0.032	1.808
Anti-SSA/Ro60kd positivity	2.560	1.137	5.069	1	0.024	12.937	1.393	120.137
Oral dryness	1.611	1.114	2.092	1	0.148	5.006	0.564	44.394
Headache	2.696	1.392	3.752	1	0.053	14.814	0.968	226.610
Myalgias	-2.973	1.275	5.432	1	0.020	0.051	0.004	0.623

## Discussion

SS is a complex disease whose diagnostic definition remains clinically challenging due to its variable manifestations and the absence of a single definitive marker [1-4,8,20,22]. The main goal of this study was to assess the correlation between minor salivary gland biopsy findings and the clinical and laboratory criteria established for SS diagnosis. Our findings confirm that patients with SS treated at the Autoimmune Diseases Outpatient Clinic at ULS Braga exhibit highly heterogeneous symptoms and disease severity.

Regarding sample characteristics, the most common age range at diagnosis (51-65 years) agrees with previous studies [1-3]. The gender distribution was also consistent with the literature, showing a female predominance of roughly 9:1 [1-3,5]. The prevalence of associated autoimmune diseases was higher than in similar studies, with 41% of patients having another autoimmune condition, most often Hashimoto’s thyroiditis, compared to 28% in other reports [2,25]. Oral and ocular dryness were the most common symptoms, seen in 78.38% and 90% of patients, respectively, aligning with existing literature [22]. However, ocular dryness was more frequent than oral dryness in our sample, a finding not consistently reported across studies [7,10,25].

Antibody positivity was higher than in previous studies: anti-SSA Ro52 in 51%, anti-SSA Ro60kd in 54%, and anti-La/SSB was also highly prevalent [18,23]. RF positivity was 40%, consistent with the literature, while ANA >1:160 was found in 64.86%, slightly lower than in some studies [6]. Articular manifestations were observed in 56.76% of patients, similar to prior reports [26].

When comparing biopsy-positive (group A) and biopsy-negative (group B) patients, notable differences appeared. In group A, RF positivity, low C3 levels, leukopenia, elevated β₂-microglobulin, ANA >1:160, positive anti-SSA, and symptoms such as aphthae, dental caries, headaches, Raynaud’s phenomenon, and arthralgia were more frequent. These findings indicate increased extraglandular involvement in this group, requiring more thorough, multidisciplinary follow-up. Literature has already associated anti-Ro/SSA and anti-La/SSB positivity with earlier disease onset, longer duration, parotid enlargement, increased extraglandular manifestations, and more severe lymphocytic infiltration [7,24].

In group B, anemia and myalgia were more common. Although no single clinical or laboratory feature is definitive for SS, the distribution of clinical and serological findings in this cohort was broadly consistent with variables included in the ACR/EULAR classification criteria: 45.9% had positive biopsies, more than half were anti-SSA positive, ocular dryness was observed in 91%, and oral dryness in 78%. However, larger multicenter studies are needed to further validate or improve the current criteria, especially considering the importance of factors like elevated ESR (67%), RF positivity (40%), ANA >1:160 (64.86%), and arthralgia (56.76%) in this sample.

To reduce false positives, it is essential to consider the entire clinical picture and conduct all recommended diagnostic tests. In suspected cases with negative biopsy results, it is important to focus on well-documented clinical features, supported by other diagnostic methods such as salivary gland scintigraphy.

It should be noted that no patients in this sample underwent genetic testing, which prevents conclusions in this area. Furthermore, only a few underwent specific tests for xerophthalmia and xerostomia, limiting the ability to fully assess diagnostic criteria. Due to SS’s clinical heterogeneity, patient management should always incorporate symptomatology and relevant laboratory findings, regardless of biopsy outcomes.

The observed correlations in this study support previously reported associations. In group A, there were strong and statistically significant links between RF positivity and anti-SSA Ro52, between anti-SSA Ro52 and ANA >1:160, and between these markers and parotid gland enlargement. In group B, strong links were found between ocular and oral dryness, anti-SSA Ro60kd positivity and anemia, and between RF positivity and anti-SSA Ro52. Previous studies have linked anti-SSA positivity to extraglandular manifestations such as anemia, leukopenia, cryoglobulinemia, vasculitis, and thrombocytopenia [20], while high ANA titers have been connected with anti-SSA/SSB positivity [6]. Conversely, the lack of correlation between RF positivity and arthralgia aligns with other findings [7].

In the multivariate analysis, anti-SSA Ro60kDa positivity was independently associated with biopsy positivity, while myalgias were associated with a lower likelihood of a positive biopsy. However, these findings should be interpreted cautiously due to the wide confidence intervals and limited sample size. The inverse association with myalgias may reflect overlap with non-glandular symptom syndromes, such as fibromyalgia-like presentations, rather than true glandular involvement.

Strengths and limitations

This study offers new insights into the relationship between salivary gland histopathology and clinical-laboratory criteria in SS, with a focus on the predictive role of anti-SSA Ro60kd antibodies. The inclusion of well-characterized patients followed over an extended period and detailed statistical analysis are strengths.

However, this study has several important limitations: (i) the small sample size (n = 37) limits statistical power and increases the risk of overfitting in multivariable analyses; (ii) the retrospective single-center design limits generalizability; (iii) inclusion was restricted to patients who underwent biopsy, introducing potential selection bias; (iv) the use of complete-case analysis may have reduced the effective sample size and introduced bias; (v) dichotomization of continuous variables may have reduced statistical sensitivity. Finally, variability in histopathological reporting limited the ability to perform detailed morphological analysis.

## Conclusions

This exploratory study identified associations between clinical, serological, and histopathological findings in SS. Anti-SSA Ro60 kDa positivity was independently associated with positive MSGB findings, suggesting a role for this antibody subtype in identifying patients with histopathologic glandular involvement, although the strength of this association remains uncertain. Conversely, myalgias were associated with a lower likelihood of biopsy positivity, highlighting clinical heterogeneity within SS.

These findings should be considered hypothesis-generating, supporting a more integrated diagnostic approach that combines serological profiling with histopathological assessment, particularly in patients with incomplete or atypical presentations. However, validation in larger, prospective, multicenter studies is required before any implications for clinical practice or diagnostic criteria can be established.

## Appendices

**Table 5:** Frequency of clinical and laboratory variables in groups A and B. SS, Sjögren’s syndrome; MSGB, minor salivary gland biopsy; ANA, antinuclear antibodies; RF, rheumatoid factor; ESR, erythrocyte sedimentation rate; OR, odds ratio; CI, confidence interval.

Analytical criteria					
Variable	Group A (n = 17)	Group B (n = 20)	Total (n = 37)	Sensitivity	Specificity
Anemia	3/17 (17.65%)	6/20 (30.00%)	9/37 (24.32%)	17.65%	70.00%
Leukopenia	6/17 (35.29%)	5/20 (25.00%)	11/37 (29.73%)	35.29%	75.00%
Low C3	5/17 (29.41%)	3/20 (15.00%)	8/37 (21.62%)	29.41%	85.00%
Low C4	0/17 (0.00%)	1/20 (5.00%)	1/37 (2.70%)	0.00%	95.00%
Positive RF	10/17 (58.82%)	5/20 (25.00%)	15/37 (40.54%)	58.82%	75.00%
Elevated β₂-microglobulin	4/17 (23.53%)	1/20 (5.00%)	5/37 (13.51%)	23.53%	95.00%
Elevated IgG	4/17 (23.53%)	4/20 (20.00%)	8/37 (21.62%)	23.53%	80.00%
ANA (>1/160)	14/17 (82.35%)	10/20 (50.00%)	24/37 (64.86%)	82.35%	50.00%
Positive anti-La/SS-B	6/17 (35.29%)	7/20 (35.00%)	13/37 (35.14%)	35.29%	65.00%
Elevated ESR	11/17 (64.71%)	14/20 (70.00%)	25/37 (67.57%)	64.71%	35.00%
Positive anti-SSA Ro52	11/17 (64.71%)	8/20 (40.00%)	19/37 (51.35%)	64.71%	60.00%
Positive anti-SSA Ro60kd	11/17 (64.71%)	9/20 (45.00%)	20/37 (54.05%)	64.71%	55.00%
Clinical criteria					
Personal history of autoimmune disease	7/17 (41.18%)	5/20 (25.00%)	12/37 (32.43%)	41.18%	75.00%
Keratoconjunctivitis	0/17 (0.00%)	1/20 (5.00%)	1/37 (2.70%)	0.00%	95.00%
Reduced tear production	14/17 (82.35%)	18/20 (90%)	32/37 (86.49%)	82.35%	10.00%
Ocular pruritus	0/17 (0.00%)	1/20 (5.00%)	1/37 (2.70%)	0.00%	95.00%
Chronic rhinitis	2/17 (11.76%)	1/20 (5.00%)	3/37 (8.11%)	11.76%	95.00%
Difficulty speaking	0/17 (0.00%)	1/20 (5.00%)	1/37 (2.70%)	0.00%	95.00%
Atrophic gastritis	4/17 (23.53%)	3/20 (15.00%)	7/37 (18.92%)	23.53%	85.00%
Dyspareunia	1/17 (5.88%)	1/20 (5.00%)	2/37 (5.41%)	5.88%	95.00%
Vasculitis	0/17 (0.00%)	1/20 (5.00%)	1/37 (2.70%)	0.00%	95.00%
Purpura	1/17 (5.88%)	0/20 (0.00%)	1/37 (2.70%)	5.88%	100.00%
Fatigue	3/17 (17.65%)	3/20 (15.00%)	6/37 (16.22%)	17.65%	85.00%
Oral dryness	14/17 (82.35%)	15/20 (75.00%)	29/37 (78.38%)	82.35%	25.00%
Oral ulcers	1/17 (5.88%)	3/20 (15.00%)	4/37 (10.81%)	5.88%	85.00%
Dental caries	6/17 (35.29%)	1/20 (5.00%)	7/37 (18.92%)	35.29%	95.00%
Ocular dryness (xerophthalmia)	16/17 (94.12%)	18/20 (90.00%)	34/37 (91.89%)	94.12%	10.00%
Paresthesia	5/17 (29.41%)	6/20 (30.00%)	11/37 (29.73%)	29.41%	70.00%
Headaches	4/17 (23.53%)	2/20 (10.00%)	6/37 (16.22%)	23.53%	90.00%
Respiratory symptoms (cough and/or dyspnea)	3/17 (17.65%)	4/20 (20.00%)	7/37 (18.92%)	17.65%	80.00%
Parotid gland enlargement/parotitis	6/17 (35.29%)	0/20 (0.00%)	6/37 (35.29%)	35.29%	100.00%
Myalgia	3/17 (17.65%)	7/20 (35.00%)	10/37 (27.03%)	17.65%	65.00%
Raynaud’s phenomenon	3/17 (17.65%)	2/20 (10.00%)	5/37 (13.51%)	17.65%	90.00%
Arthralgia	11/17 (64.71%)	10/20 (50.00%)	21/37 (56.76%)	64.71%	50.00%
